# The Global Burden of Disease Study tuberculosis estimates from the Institute for Health Metrics and Evaluation

**DOI:** 10.1093/ije/dyae122

**Published:** 2024-09-26

**Authors:** Hmwe H Kyu, Jorge R Ledesma, Christopher J L Murray

**Affiliations:** Institute for Health Metrics and Evaluation, University of Washington, Seattle, WA, USA; Department of Health Metrics Sciences, School of Medicine, University of Washington, Seattle, WA, USA; Institute for Health Metrics and Evaluation, University of Washington, Seattle, WA, USA; Department of Epidemiology, Brown University School of Public Health, Providence, RI, USA; Institute for Health Metrics and Evaluation, University of Washington, Seattle, WA, USA; Department of Health Metrics Sciences, School of Medicine, University of Washington, Seattle, WA, USA

**Keywords:** GBD, tuberculosis estimation, methodology, collaboration, HIV-TB coinfection

In their opinion piece, Dodd *et al*. outlined the Independent Advisory Committee TB deep dive panel’s recommendations for tuberculosis (TB) estimation for the Global Burden of Disease study (GBD).^1^ On behalf of the GBD TB research team, we have provided our responses below.

Regarding the first recommendation, which emphasizes the need for clear, self-contained method explanations and reproducibility, we align this with the overall goal of collective research teams at the Institute for Health Metrics and Evaluation (IHME): to develop user-friendly, efficient and robust modelling tools for IHME, GBD collaborators and institutional partners. The ongoing efforts of IHME’s research teams seek to enhance the clarity and reproducibility of GBD methods, by allowing for consistent replication of findings across teams and making complex data modelling more accessible to a broader user base, thereby promoting transparency and verifiability in global health research.

The second and third recommendations call for strengthening dialogue with the World Health Organization (WHO) and refining methods to better link TB estimates with country data. Efforts to foster stronger collaboration between IHME and WHO, to address data quality issues and produce more robust TB estimates, are under way. It is worth noting that estimates may vary when data quality and coverage are limited, as different methodologies are used to address these limitations. When national TB data quality and coverage are high, the differences in estimates between the two groups should be minimal. Given the limitations of data sources, such as notifications and vital registration data, the measurement of incidence, prevalence and mortality may not be perfect. Hence, we used our Bayesian meta-regression tool, DisMod MR 2.1,^2^ to conduct statistical triangulation of all available data sources, aiming for a more accurate assessment than relying on a single data source. DisMod estimated lower incidence than notified cases given vital registration deaths in certain countries. Notably, less than 50% of new TB notifications in these countries were bacteriologically confirmed,^3^ implying the possibility of overdiagnosis^4^ and over-reporting. Aligning estimates with notification data might address some critiques but may not yield accurate estimates for monitoring progress towards global targets. Addressing these challenges and crafting practical solutions require a diverse range of disciplinary and experiential knowledge.^5^ It is crucial for IHME, WHO and national TB programmes to collaborate in refining strategies to resolve data discrepancies and enhance data quality, possibly through establishing comprehensive data collection systems with standardized tools and developing innovative methods to correct for underlying biases in the data.

**Figure 1.**
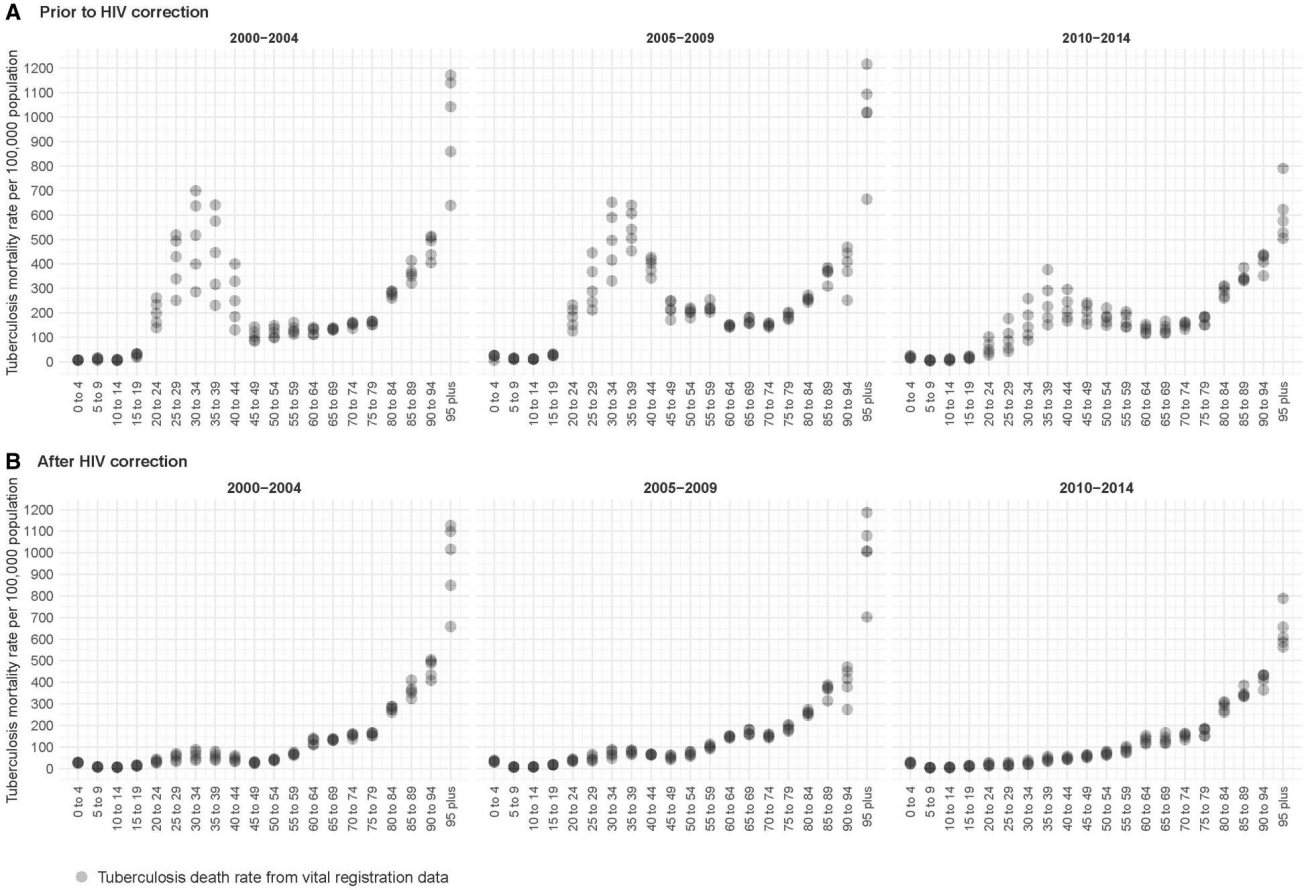
HIV misclassification correction to tuberculosis vital registration data in South Africa: before correction (A) and after correction (B). Black points are death rates per 100 000 population due to tuberculosis without HIV co-infection from vital registration data in South Africa. Included in the figures are age-specific tuberculosis death rates from 2000 to 2014. Panel (A) visualizes tuberculosis death rates prior to HIV misclassification correction, whereas panel (B) visualizes tuberculosis death rates after HIV misclassification correction. This adjustment is performed due to evidence that HIV deaths are misassigned to other underlying causes of deaths, including tuberculosis

The last recommendation suggests updating or justifying the rationale for equal duration of disease by HIV status and sex. Given the scarcity of empirical HIV-TB duration data, we model all-form TB without differentiating by HIV status, using all-form TB duration estimates as priors along with age-sex-specific TB mortality, prevalence and incidence data to derive age-sex-specific all-form TB duration estimates. We then divide the all-form TB incidence and prevalence estimates from DisMod into HIV-positive and HIV-negative categories, according to the proportions of TB patients testing positive for HIV. Accurate quantification of HIV-TB duration necessitates considering a variety of factors. TB disease progresses faster in people living with HIV (PLHIV), leading to earlier symptoms and quicker detection, though the persistence of this detection pattern in settings with limited diagnostic access remains uncertain.^6^ Furthermore, considering the definition of duration^7^—from onset to cure or death—evidence suggests that PLHIV may benefit from longer TB treatment regimens.^8^ Whereas disease duration can often be simplified by dividing prevalence by incidence when rates of incidence, cure and death are stable, this becomes significantly more complex for HIV-TB co-infection in the context of known limitations in notifications and prevalence data along with the frequent misclassification of deaths from HIV-positive TB as HIV-negative TB. Vital registration data indicate that HIV deaths were commonly mis-assigned to HIV-negative TB,^9^ necessitating corrections for misclassification in vital registration data in high HIV prevalence countries, such as South Africa (Figure 1). Concerns also arise from using HIV-TB data from TB prevalence surveys due to high HIV testing refusal rates and reliance on self-reported HIV status.^10^^,^^11^ These issues collectively highlight the critical need for concerted collaborative efforts to enhance data quality and improve TB burden estimates.
